# Exploring ethnic minority and underserved groups’ experiences of the National Health Service Cardiovascular Disease Health Check uptake in North East England: applying a behavioural insights, qualitative approach

**DOI:** 10.1136/bmjopen-2024-096500

**Published:** 2025-09-21

**Authors:** Sophia Margarita Brady, Joe Chidanyika, Karen Verrill, Jennie Sofia Portice, Steph Scott, Julia Newton

**Affiliations:** 1 NIHR Applied Research Collaboration North East and North Cumbria, Gosforth, UK; 2 Population Health Sciences Institute, Newcastle University Faculty of Medical Sciences, Newcastle upon Tyne, UK; 3 Health Innovation North East and North Cumbria, Newcastle upon Tyne, UK

**Keywords:** Cardiovascular Disease, Qualitative, Focus Group, Health Check, Behavioural Insights

## Abstract

**Abstract:**

**Background:**

The North East of England has the lowest healthy life expectancy and the highest health inequalities of any region in England. The conventional model, whereby we ‘expect’ individuals to be motivated to attend a ‘healthcare setting’ to undergo cardiovascular disease (CVD) health checks every 5 years has low levels of uptake, with populations most at risk frequently failing to engage.

**Objectives:**

The objective of this study was to gather behavioural insights into the barriers/challenges that limit engagement with the current NHS CVD Health Checks.

**Methods: design, setting, participants and outcomes:**

Drawing on a Behavioural Insight approach, 7 qualitative focus groups with members of ethnic minorities and underserved groups (n=45 participants) were conducted to understand barriers and challenges to uptake of NHS CVD Health Checks in one region in North East England (Middlesbrough). Data were analysed using a Behavioural Insights approach, applied to establish key themes, barriers and enablers.

**Results:**

Our findings identified that underserved communities in North East England find engaging with NHS CVD Health Checks challenging due to issues related to access, understanding and attitudes. Communities identified that harnessing relationships with existing community champions would raise awareness and confidence in engaging. Making services accessible where communities gathered, while also increasing understanding and knowledge, was also recognised as key to engagement.

**Conclusions:**

Our study suggested that despite there being substantial barriers to engagement with NHS CVD Health Checks, novel methods encouraging uptake may be effective to address the significant health inequalities seen in deprived communities. Ensuring a co-developed and co-delivered approach to CVD risk reduction with underserved communities, together with social marketing campaigns to raise awareness about the importance of CVD, and why reducing its risk is so important, is key to success.

**STRENGTHS AND LIMITATIONS OF THIS STUDY**

- Strengths of this study include the framework analysis using the widely used and validated Behavioural Insights approach.
- We included a diverse group of participants from a wide range of underserved populations and ethnic minority groups to obtain a range of viewpoints from both NHS patients and NHS professionals.
- Limitations include the modest sample size of 45 participants in focus group discussions.
- Another limitation is that the study was conducted in populations within Middlesbrough, and findings may not be transferable to other ethnic minority and disadvantaged groups living in other areas of the country. Barriers and facilitators may be region-specific, and studies must be conducted in other areas to ensure interventions are appropriate for the population of interest.

## Introduction

 Cardiovascular disease (CVD) is the leading cause of death worldwide, with around 7.6 million people in the UK living with CVD, and 25% of UK deaths caused by CVD.^1^ CVD and its related comorbidities have a significant economic cost, with estimations of healthcare costs of over £7 billion, and wider economic costs of almost £16 billion in 2019.^1 2^ The prevalence of CVD varies within different groups, with people living in the most deprived regions of the UK being four times more likely to die prematurely from CVD than those living in the least deprived regions.^2^,^4^ However, CVD is preventable, and there are opportunities to modify risk factors and raise awareness of the potential dangers of CVD, which have been highlighted as targets by the UK government.^2^ CVD prevention is therefore a healthcare and policy priority.

In the UK, adults between 40–74 years should be invited to a free NHS CVD ‘Health Check’ every 5 years.^5^ This national scheme is publicly funded, and its aim is to aid the screening, prevention and raising awareness of CVD.^6^ However, previous research and reports have demonstrated variable uptake of the NHS CVD health check among the general population,^7 8^ with average UK attendance being only 44% of those invited in 2022 according to a recent systematic review.^7^ Furthermore, multiple studies have found significantly lower levels of attendance within populations experiencing high levels of deprivation,^7^,^9^ demonstrating populations most at risk frequently fail to engage.^10 11^ Furthermore, concerns have been raised among commissioners and providers of these NHS CVD Health Checks relating to how the delivery and practical implementation of these programmes risk increasing health inequalities if implemented in a way which does not systematically prioritise equity of access, outputs and outcomes, especially among underserved population groups.^3 10^

The Northeast and North Cumbria (NENC) has the lowest healthy life expectancy and highest health inequalities of any region in England.^3 12^ Men and women not only have lower life expectancy than the national average, but they also spend a larger proportion of their shorter lives in poor health.^3 12^ Specifically, Middlesbrough has one of the lowest life expectancies in England, with >50% of the population living in the most deprived quintiles in the country, and it has the highest rate of years of life lost for males and females in NENC. CVD contributes to over 10% of the 3.9 year gap, and over 16% of the 3.8 year gap in life expectancy between Middlesbrough and England in 2020/21 in males and females, respectively. Furthermore, between the most and least deprived areas within Middlesbrough, CVD contributes towards 20% of the 11.1 year gap for males and 15% of the 8.8 year gap in life expectancy for females.^12^,^14^ Studies have shown that areas with higher levels of inequalities and with disadvantaged populations have reduced uptake of healthcare and prevention services, with NHS CVD Health Checks not an exception.^7 11 15^ Although invitation methods for NHS CVD Health Checks have changed over time, previous research has emphasised the need for invitation methods to be enhanced to ‘drive uptake while reducing health inequalities’,^16^ as disadvantaged population groups continue to have poor uptake of NHS CVD Health Checks.^7^

Despite this, no studies have specifically explored the perspectives and experiences of NHS CVD Health Checks in underserved populations within Middlesbrough, particularly those who fall within the Core20PLUS5 population. Core20PLUS5 is an NHS England target population, non-exclusively including the most deprived 20% of the population, ethnic minority communities, refugees and those at risk of certain clinical conditions. Groups that fall within this definition will vary geographically depending on the protected characteristics of the region. Therefore, the aims of this study were to use a qualitative behavioural science insights approach to explore the experiences, facilitators and barriers of Core20PLUS5 groups (hereby referred to as ‘underserved groups’) engaging with NHS CVD Health Checks (and other NHS health checks) in Middlesbrough. This study was conducted in collaboration with the Health Innovation Network, and a requirement of funding was to co-design some local interventions with communities. Therefore, secondary aims were to subsequently co-design potential solutions to facilitate greater engagement with NHS CVD Health Checks and share learnings to accelerate uptake of NHS CVD Health Checks locally, regionally and nationally, for underserved groups. Designed solutions will be discussed in an implications for policy and practice section.

## Materials and methods

Seven qualitative focus groups were conducted with members of underserved groups in Middlesbrough, North East of England, between May and July 2023. Middlesbrough was chosen as the region for this study due to its having particularly high levels of deprivation and rates of CVD, as well as an ethnically diverse population. These unique characteristics make Middlesbrough an ideal target area to capture a range of insights from populations who could benefit most from NHS CVD Health Checks, as it is distinctive within both the North East and the rest of England. Focus groups were conducted due to the rich qualitative data they provide through group interaction and discussion,^17^ and to increase uptake and reduce burden, as participants were from underserved groups and may have felt more at ease in a group environment.

Eligible participants were purposively sampled according to the following criteria:

- Adults (≥18 years),
- Residing in Middlesbrough
- From a range of underserved groups (ie, Core20PLUS5). Following England’s definition of COREPLUS5 and using Middlesbrough as a context for this work, we included participants from the most deprived communities (CORE20) using the Index of Multiple Deprivation. This included white British groups who were economically disadvantaged (ie, residing in areas of high deprivation). We also included (PLUS) populations likely to experience poor health but may not be represented by the CORE20. This included: migrants, asylum seekers, South Asian men and women, Black African, Eastern Europeans.
- In addition, as we were using a purposive sampling approach, we decided to include a group of professionally qualified migrant doctors (REPOD, Resettlement Programme for Overseas Doctors and other health professionals). This group was included because they fit many of the above criteria (eg, migrant, refugee, ethnic minority), and could add the perspective of a medical professional having worked in multiple contexts.

These groups and the broad inclusion criteria were chosen to capture a range of perspectives from these groups. No screening was required as the inclusion criteria were clear, and community leaders acted as gatekeepers and were key in recruitment. Recruitment was aided by existing partnerships and relationships between the research team and community leaders, with participants invited to take part through word of mouth and email invites. All participants received a study information sheet and provided written consent prior to taking part. Written study materials were made available in several languages, including Arabic and Urdu, and we collaborated with community leaders to ensure that focus group venues and times were selected to ensure accessibility for potential participants. Specifically, venues were within participant communities for convenience, with sessions held at mosques, women-only gyms and community centres. A total of six focus groups were delivered face-to-face, and one focus group was held virtually on Microsoft Teams and was conducted by two members of the research team (JC/KV/SMB/JSP, 3 female, 1 male). All researchers were experienced in conducting focus group discussions and qualitative research, and none had existing relationships with the participants. The study was granted ethical approval by Newcastle University’s FMS Ethics Committee (Ref: 32236/2023). To thank them for their time, all participants were provided with a £15 gift voucher.

A semi-structured topic guide (online supplemental figure 1) was used by the research team to guide focus group discussions. Specifically, focus groups explored participants’ interactions with health services in general, knowledge of CVD and importance of NHS CVD Health Checks, as well as their personal experiences and understanding of potential barriers and facilitators to attending such Health Checks. All focus groups were audio-recorded and transcribed verbatim by staff at Health Innovation NENC, with observational field notes maintained in a research diary.

Transcripts were analysed using Framework analysis structured according to the EAST framework, developed by the Behavioural Insights Team^18^ (table 1). We therefore followed the five core stages of this approach outlined by Ritchie, Spencer and O’Connor,^19^ moving back and forth between stages throughout the analysis: (1) Familiarisation, (2) Identifying a thematic framework, (3) Indexing, (4) Charting and (5) Mapping and interpretation.

**Table 1:** Stages in the development of the EAST framework applied to NHS cardiovascular disease Health Checks

*Defined outcome*	Increased uptake of overall CVD health checks Better target audience knowledge of CVD as a condition Better comprehension of key uptake barriers and enablers Suitable alternative settings for intervention delivery
*Project context*	High levels of deprivation in certain Middlesbrough wards Poor health check uptake for marginalised groups Good engagement through the pandemic from underserved groups for COVID-19 vaccinations
*Building interventions*	Apply the EAST framework during focus groups Framework analysis of transcripts to deduce emerging themes Hold debrief sessions with community leaders to discuss emerging themes
*Testing, learning and adapting*	Engage with wider stakeholders, including service commissioners Use feedback from community leaders and stakeholders to co-design interventions Develop a targeted social marketing campaign based on co-designed feedback Pilot developed interventions with endorsement from key stakeholders

CVD, cardiovascular disease.

The EAST framework uses a Behavioural Insights approach to explore service user experiences of interventions and has been used previously in health settings to understand and potentially influence behaviour to improve outcomes for example, drug and alcohol support, smoking cessation services and physical activity.^20 21^ The framework acronym stands for Easy, Attractive, Social and Timely, and these were applied to the research question and topic guide (table 2, online supplemental figure 1). By applying the EAST framework to our data, we identified and mapped core factors that related to the four pillars of the framework, and we were able to use this organising structure as a tool through which to explore participants’ lived experiences of accessing NHS CVD Health Checks. In keeping with accepted conventions of qualitative Framework Analysis, we also remained cognisant throughout of any other thematic patterns or trends in the data that could have been of importance to participant experiences and to our overall interpretation of the data.^19 22^ Five members of the research team (JC/SMB/JSP/SS/KV) conducted qualitative analysis and populated the framework.

**Table 2:** Components of the EAST framework in relation to this study

*EAST framework component*	*Framework narrative*	*Factors assessed in focus groups*
*Easy*	How easy is it to access NHS CVD health checks or understand the condition and its risk factors?	Interactions with health services Ease of these interactions What would make them attend health checks? How are people invited? Where is it ideal for health checks to be conducted, and the best way to be invited?
*Attractive*	How attractive is it to prevent CVD?	Is CVD prevention important? Would you take up health checks? How can CVD prevention be marketed?
*Social*	What are the positive or negative views of participants and their communities on taking up NHS CVD health checks?	What support is needed for communities to access health checks? What kind of invitations would work for your communities? What information is helpful for communities to share about CVD? How can CVD Health Check services suit or improve your lifestyle?
*Timely*	How can health-seeking behaviour change to improve uptake? When is the best time to intervene?	Which behavioural change models can increase uptake? How can behaviour change be sustained? What costs are involved in accessing services? Any windows of opportunity (tagging on family CVD history) to influence health-seeking behaviour? Nudge?

CVD, cardiovascular disease.

### Patient and public involvement

This study had public involvement in the study design, recruitment and dissemination. Community leaders aided recruitment of study participants and helped inform focus group topic guides. In addition, local community leaders and public health commissioners were invited to a debrief session to disseminate study results and co-design innovative solutions.

## Findings

Seven focus group discussions, lasting approximately 90 min each, were held with a total of 45 participants (age range=24–72 years, 27 females, 18 males) from four underserved/Core20PLUS5 target communities across Middlesbrough, representing South Asians, Black Africans, White underserved groups, as well as REPOD healthcare professionals. Our findings presented below are organised according to the EAST framework and include quotations to provide rich, contextually relevant and faithful accounts of the experiences of participants in our study. All data were coded anonymously to ensure that participants were not identifiable from their accounts. Thus, very little additional demographic information is reported here in order to safeguard our participants and thus protect anonymity and confidentiality.

### Theme 1: making NHS CVD Health Checks easy

Some can’t speak, so if they go to the doctor what can they say…

Black African Focus Group

All seven focus groups shared how difficult it is to not only get a General Practice (GP) appointment for overall health issues, but also the difficulty in getting specific NHS CVD Health Check appointments. Discussions focused on what prevented attendance and how barriers could be removed. Multiple communities highlighted that the ‘hassle factor’ of taking up NHS CVD Health Checks could be reduced, and how CVD as a condition and its language could be simplified to increase accessibility to all. For some, issues accessing GPs for appointments were perceived as related to their ethnicity, especially by those seeking asylum, who felt they were ‘blamed’ for congesting the health system. Waiting times for every group were an issue, with some waiting for more than a year to be seen for key appointments. Further, participants within certain occupations, for example, shift workers, found getting appointments particularly challenging. These participants felt that if services could be brought to them within their ‘workplaces’, this could improve engagement:

They don’t really have a social life, that’s they go home, go to sleep, get up in the morning, pick their taxi keys up, go to the rank, sit there, talk and work, that’s it.

South Asian Men’s Focus Group

Language was a barrier consistently referred to by participants whose first language was not English, even when attending appointments with a translator. Participants cited problems in both understanding their healthcare professionals and not having the confidence to attend appointments altogether due to language barriers. Language barriers were closely linked to the notion of health literacy. For example, for those who spoke the English language, terminology used by health professionals was sometimes unhelpful, with health professionals frequently using medical jargon, with participants referring to this jargon as *"Doctor Speak’*’. Translation services were also requested for most non-English speakers, with many of these requests not having any positive consequences. However, even for those who used translation, they still encountered barriers and questioned the accuracy of what was translated.

I think one of the biggest barriers in some patient communities, and mostly from Asian, is the language problem cos most find it difficult to have people in their family who can speak English.

Black African Focus Group

Knowledge of what exactly CVD is, and what an NHS CVD Health Check consists of, was discussed at length in focus groups, with few participants having any awareness unless they had a health check themselves. The REPOD healthcare professionals echoed these concerns and highlighted the need for increased awareness, particularly in underserved communities. REPOD focus group participants suggested that education was needed to improve health literacy for their communities as an effective empowerment tool, to improve engagement and make it easier to attend health checks.

When you say health check, does that just mean like a BP check or?

White British Focus Group

….the important responsibility is for the person as a person, okay to care about himself, to know what he has, and to know what to do or not. He has to educate himself, and I would say that’s the most important…

REPOD Professionals Focus Group

### Theme 2: making NHS CVD Health Checks attractive

It is just the way you get treatment from the GP and the reception staff. That’s why it puts me off.

White British Focus Group

Participants explored the need for more nuanced cultural respect and for gender-appropriate services. Some women reported having to change their GP practices due to a lack of gender-specific cultural needs. Staff attitudes towards patients resulted in negative experiences for some, especially new migrants seeking asylum. Although GP staff were valued, there were suggestions of the need for cultural competence training and for better staff attitudes to ethnic minority patients. Some people viewed receptionists as gatekeepers who were a barrier to either getting an appointment. For patients with other health needs such as learning disability or mental ill health, parity of esteem was felt to be important. Furthermore, there were trust issues from some communities who felt the practice staff were dismissive. Some experiences shared suggested that accessing healthcare for some became unattractive even when very unwell, with participants reporting they preferred to attend Accident and Emergency departments.

Sometimes, you know, Asian ladies, prefer female doctors. So we prefer that we need a female doctor, and sometimes they don’t and sometimes they listen, but sometimes they say no, we have only the men.

South Asian Women’s Focus Group

Then, because of the lack of customer service, they get put off, and then when they get an invitation, it feels like now you need me…

Black African Focus Group

Meanwhile, shared decision making was identified as much needed as most participants, especially asylum seekers, felt some tests were coerced and not consented to, or at least explained properly. Participants felt patients from ethnic minorities should be more involved in their own CVD care, with better culturally appropriate shared decision-making guides developed. Some participants strongly felt discriminated against, not only when accessing NHS CVD Health Checks, but healthcare in general.

They will just take blood from you without your knowledge of what they are testing for then the next thing is you are told about your HIV status and it’s wrong

South Asian Men’s Focus Group

### Theme 3: social aspects of NHS CVD Health Checks — communities and outreach

It would benefit everybody. If it was in the community people would go there…

South Asian Men’s Focus Group

The sentiment of tokenistic engagement from health services for ethnic minority participants was evident, with communities describing the feeling of becoming over-researched but underserved. This further compounded mistrust of the health system and poor engagement with some services. Participants were keen to have feedback from studies and influence service provision for their neighbours. Other groups, such as underserved white communities, felt they were undeserving of better healthcare and a drain to the system:

So this (research project) is not just a paperwork exercise is it?

Black African Focus Group

Sometimes they might think I’m whinging and being a burden by going, so you just keep it to yourself…

White British Focus Group

It’s not been exactly said that but we seem to be moving in a direction where you have a sort of undeserving poor who shouldn’t have the same access to health services as people who have better resources.

White Mixed Focus Group

Social aspects were also seen by participants as key targets for increasing NHS CVD Health Check uptake. For example, communities that felt behaviour change is supported within community settings will see tangible and sustainable positive health outcomes. There was clarity from participants that a community development outreach approach was an attractive option in addition to the traditional GP/secondary care approach to CVD health improvement. Services closer to their doorstep were not only conducive, but would enable uptake to scale and engage others, as there is a big sense of community willpower to improve health. Community outreach and social marketing approaches for NHS CVD Health Check uptake were among the suggestions due to their convenience.

Because of work it was accessible via the radio wasn’t it, the centre there. So I had my health heart check done there but tis not something that’s on the forefront of my mind

South Asian Men’s Focus Group

A further suggestion within focus groups was to use existing community leaders and/or volunteers as health champions and role models within communities, to raise awareness about NHS CVD Health Checks. Voices within focus groups felt their volunteering capability was underused or harnessed. Some were migrants who were health professionals who couldn’t work but felt that through health championing they could ‘give back’. Some were keen to use volunteering to boost their own employment opportunities. For example, the REPOD programme was seen as an innovative approach to harness ‘wasted professionals’ not allowed to work due to their migration status, as some asylum seekers were highly qualified health professionals in their home countries.

There are a lot of nurses and doctors here just sitting studying doing nothing. Most of us are willing to do this job voluntarily…

Black African Focus Group

### Theme 4: timeliness of NHS CVD Health Check appointments and treatment

You have to ring in the morning, and it delays you and it puts you off contacting them.

Black African Focus Group

Long waiting times for appointments were perceived as off-putting for many, impacting their proactive ownership of their health and well-being. In terms of the designated times required to call GP surgeries for an appointment, many female participants felt that this clashed with other responsibilities, such as doing the school run. This was reiterated by female participants at multiple focus groups, and there was a consensus that the health and welfare of their children and families was more important than their own. Despite this, many participants recognised the importance of attending regular NHS CVD Health Checks to maintain their own health and continue providing for their families. There was a consensus for either walk-in sessions to be allowed or afternoon appointment calling times to also be introduced:

Surgery time is between 8–9, and school kids time is between 8–9. Obviously first thing in the morning mothers think, we will drop the kids first…

South Asian Women’s Focus Group

While many participants demonstrated commitment to getting NHS CVD Health Checks, there was a vocalised need for more flexible appointment times, especially for those working unsociable hours or with parental responsibilities. Walk-in centres were suggested as an alternative:

Years ago they had walk in centres. It doesn’t matter how many hours you are waiting there because you are sure someone will check you…

South Asian Men’s Focus Group

My husband drives a taxi and if he needs appointment, and there is 1 hour, and if they give an appointment at an odd time, obviously he needs to work.

South Asian Women’s Focus Group

## Discussion

### Summary of findings

This study used qualitative focus groups, underpinned by a Behavioural Insights approach, to obtain a greater understanding of the experiences, facilitators and barriers of ethnic minorities and underserved groups engaging with NHS CVD Health Checks as an intervention to prevent CVD mortality and risk factors in Middlesbrough. Overall, the main barriers to NHS CVD Health Checks were around ease and flexibility of access, language barriers, lack of knowledge, discriminatory staff attitudes and lack of shared decision-making. There was a strong feeling around the need for health services to be more considerate of those from different ethnicities, cultures and backgrounds, and for health services to recognise and accommodate the specific needs of these underserved communities. However, although generally perspectives were negative, with numerous barriers cited, participants were hopeful, raising suggestions around the use of community role models and health champions to help increase CVD knowledge and health check engagement, and co-design solutions specifically for underserved groups within Middlesbrough and beyond. There was an overarching belief that, individually, their voices were not heard, but through health champions, community leaders or local commissioners, there was the potential for change within the healthcare system.

The barriers given toward NHS CVD Health Checks were comparable to findings from previous studies.^15 23 24^ One systematic review from 2018 examined the barriers and facilitators towards health checks within different communities in Europe and found that practical reasons such as lack of time, difficulties getting an appointment and accessibility of health checks were some of the major barriers to attendance.^24^ de Waard *et al*^24^ also highlighted that socioeconomic status, ethnicity and education level were observed as barriers or facilitators for people to participate in a health check for cardiometabolic disease. Some of the more generic recommendations to increase NHS Health Check uptake, such as provision of evening or weekend appointments, were a consistent finding with studies conducted in other areas of the UK.^15^ However, the novelty of this study lies in the population-specific facilitators, barriers and recommendations found within ethnic minority and disadvantaged groups in Middlesbrough. With Middlesbrough being a particularly deprived region, with higher-than-average CVD rates than the rest of the UK, this highlights the importance of this study and emphasises the impact of our findings. A further review by Harte *et al*^23^ explored non-attendance to NHS CVD Health Checks and found similar reasons for non-attendance to this current study, including lack of knowledge, time constraints and difficulty in access. Our findings build on the results of these studies and others,^25^,^27^ and add the perspectives from underserved population groups whose views are commonly missed in large-scale research.

### Implications for policy and practice

Middlesbrough has some of the highest prevalence of CVD in England and the poorest health outcomes. Understanding why communities have not historically engaged with the conventional ‘clinic’ based model for addressing CVD risk is vital if improvements in CVD mortality and morbidity are to be delivered. Assumptions can sometimes be made by those commissioning services without direct engagement with those communities who would most benefit. This study has shown that working with communities can help promote greater understanding (for both communities and for commissioners), which could ultimately lead to greater engagement.

This study highlights the need for imminent action from health professionals and policymakers. For example, there is an urgent need for investment in local social marketing campaigns to raise the awareness of populations in the North East of England of the risks of CVD, and to provide information on the NHS CVD Health Check programme. Our study and others show that despite the NHS Health Check programme being in place for over 15 years, many people within the North East and nationwide, and particularly those living in areas of deprivation, still do not have awareness of their existence or knowledge of the importance of CVD risk prevention.^9 15 16 23 25 27^ Investment in these campaigns, as well as more population-specific bespoke invitation methods^15 25 27^ may lead to increased NHS Health Check uptake, and consequently long-term reduction in local and national healthcare costs from CVD. Furthermore, utilisation of existing community settings, training other health professionals to conduct NHS Health Checks and the need for flexible GP appointments are urgent regional policy suggestions from this study and others to increase NHS Health Check attendance within those at greatest risk and subsequently reduce CVD disease burden.^11 15 23^

As a consequence of this research, a debrief session with key influential community leaders and public health commissioners was carried out, where findings were fed back, views were sought and potential local solutions considered. This session led to the development of innovative interventions to facilitate greater uptake of NHS CVD Health Checks within Middlesbrough. Co-designed interventions, which took place led to tangible outcomes, such as the recording and dissemination of regional social marketing campaigns via a debrief video fronted by key community leaders and researchers with a narrative of why NHS CVD Health Checks are important and where to get them. Both participants and community leaders stressed that an important element of intervention development is not to assume what will support communities, and interventionists must take time to learn what will specifically enhance a population’s ability to engage. There was a huge appetite for health check delivery models outside of a GP practice, through the utilisation of existing social capital and community assets. Consequently, a community outreach programme of mobile NHS CVD Health Checks was established, with health checks taking place at different locations, such as local football clubs, university clinics and community hubs. This targeted community outreach approach has been successfully implemented in other regions to increase NHS CVD Health Check uptake among disadvantaged and ethnic minority groups.^7 28^

Lastly, cultural competence was a big priority for focus group participants. Further co-designed interventions created as a consequence of this research included the development of a bespoke training package to upskill health champions and community leaders as a trusted workforce to increase community-based health checks, as well as the co-creation of tailored health heart check resources, competency frameworks for outreach providers, and bespoke staff-focused cultural competency training.

Other studies have developed different interventions, such as a personalised CVD ‘Risk report’ of health check results to give to NHS Health Check attendees, which had positive feedback from participants.^5^ However, it would be a challenge to make risk reports available in other languages and at non-GP settings, limiting the transferability of these recommendations to this study population. In contrast, interventions developed as part of this study were co-designed with the communities themselves and may therefore lead to greater sustained long-term behaviour change within those underserved communities of Middlesbrough with the greatest need.

### Strengths and limitations

Strengths of this study include the framework approach to analysis using the widely used and validated Behavioural Insights approach.^18^ We included participants from a wide range of underserved groups to obtain a range of viewpoints. A further strength is that focus groups were conducted within settings familiar to, and local to, participants, which ensured they were comfortable and confident to give their true opinion, which enhanced the value of findings.

Nevertheless, this study was not without its limitations. Participants were from diverse backgrounds to capture a range of viewpoints. However, as the Black and minority ethnic population of Middlesbrough has now grown beyond 17% of the total population,^29^ a larger sample size could have enhanced the validity of our findings. Second, community leaders aiding in recruitment may have known participants, potentially creating selection bias in the recruitment process. However, these community leaders were essential for us to gain access to underserved groups. Furthermore, due to the poor quality of recordings and the use of translators in some focus groups, transcribers were unable to confidently attribute quotes within transcripts to specific participants. We therefore did not have this information in the transcripts, nor is it included in the quotes in the analysis. This could have been mitigated with better equipment or the use of video recordings rather than audio. As with any qualitative work, despite the depth and richness of the data, it has limited generalisability. Future studies could include a quantitative element to study if barriers and facilitators have differing importance among different groups, in order to subsequently design more personalised interventions to target these subgroups more specifically. Nevertheless, to our knowledge, this is the first study qualitatively exploring the perspectives of underserved groups in England towards NHS CVD Health Checks.

### Conclusion

In this qualitative analysis of the perspectives of ethnic minority and underserved populations within Middlesbrough, we found the barriers to attendance of NHS CVD Health Checks were numerous and varied within different groups. Our novel findings identified numerous small-scale but effective interventions which led to tangible increases in NHS Health Check uptake within the region. However, we also identified that further nationwide interventions and considerable financial investment are needed to increase CVD Health Check uptake within other underserved groups nationwide, and that interventions to increase uptake of NHS prevention programmes need to be innovative and population-specific to lead to noticeable reductions in CVD risk and mortality. The government and policy makers urgently need to invest in NHS CVD Health Checks and develop CVD prevention strategies to reduce the burden of CVD on both individuals and an already overstretched NHS.

## Supplementary material

10.1136/bmjopen-2024-096500 online supplemental file 1

## Data Availability

All data relevant to the study are included in the article or uploaded as supplementary information.
