# Supplementary material for: Exploring ethnic minority and underserved groups’ experiences of the National Health Service Cardiovascular Disease Health Check uptake in North East England: applying a behavioural insights, qualitative approach
Source: BMJ Open. 2025 Sep 21;15(9):e096500. doi: 10.1136/bmjopen-2024-096500 (PMC12458740; doi:10.1136/bmjopen-2024-096500)
Supplement: online supplemental file 1 [file bmjopen-15-9-s001.docx]

***Supplementary Figure 1: Interview Topic Guide***

*Initial questions:*

- Are you aware that cardiovascular disease is the main cause of death or disability in England and here in Teesside? (Deliberate closed question to set off discussions)
- What are the barriers to attending health checks in order to reduce you or your community’s CVD risk? (Open question)
- How do you currently engage with the health services / GP practices, to improve or maintain your heart health?
- If we are to help improve your uptake of CVD health checks, what approaches will work for you and your community, to engage better in preventing poor heart health?

*Suggestion for change:*

So we are here today to talk a little about your thoughts and experiences around the health system, we are interested in the general but also some more specific experiences around NHS Health Check.

So first let me ask you:

**1.)** Have you had many interactions with the health services? How were these experiences?

If negative, why? If positive, why? If none why?

**2.)** Have you heard of cardiovascular disease? Do you know what it is?

**3.)** Have you ever received any kind of information about/been invited for an NHS/cardiovascular disease health check (to be had every 5 years)?

(If applicable) How did you receive your invitation (txt, GP, letter?).

What did you think of it? If nothing, do you wish you had received it? If yes, How, what format?

**4.)** Have you attended an NHS CVD health check?

Why? Why not?

What was the experience like?

Have any of your family members/ friends attended?

**5.)** What do you know about the risk factors in relation to cardiovascular disease?

Does this concern you? Why/why not?

**6.)** Are there any specific things that make you more/less inclined to attend such health checks?

Please elaborate.

**7.)** Some people find attending these sorts of health checks difficult for a variety of reasons. Have you experienced this? What were the reasons?

**8.)** If there is a way you think the health service could help you and your family/ community be more inclined to attend health checks what do you think that would be?
