# Supplementary material for: Exploring ethnic minority and underserved groups’ experiences of the National Health Service Cardiovascular Disease Health Check uptake in North East England: applying a behavioural insights, qualitative approach
Source: BMJ Open. 2025 Sep 21;15(9):e096500. doi: 10.1136/bmjopen-2024-096500 (PMC12458740; doi:10.1136/bmjopen-2024-096500)
Supplement: Supplementary file 2 [file bmjopen-2024-096500.reviewer_comments.pdf]

PEER REVIEW HISTORY

BMJ Open publishes all reviews undertaken for accepted manuscripts. Reviewers are asked to complete a checklist review form and are provided with free text boxes to elaborate on their assessment. These free text comments are reproduced below.

ARTICLE DETAILS

Title (Provisional)

Exploring ethnic minority and underserved groups' experiences of the National Health Service Cardiovascular Disease Health Check uptake in North East England: applying a behavioural insights, qualitative approach.

Authors

Brady, Sophia Margarita; Chidanyika, Joe; Verrill, Karen; Portice, Jennie Sofia; Scott, Steph; newton, Julia

---

VERSION 1 - REVIEW

**Reviewer**	**1**
**Name**	**Marino, Katherine**
**Affiliation**	**Keele University School of Medicine**
**Date**	**23-Nov-2024**
**COI**	**None**

Thank you for your work on this project. It is a great topic and well-written paper.

General Comments

Percentages are best kept to whole numbers, without decimal places, to help the piece read better- personal opinion and will leave this to authors discretion.

Introduction

Good introduction, sets the topic up well.

Line 3- UK shouldn't be an abbreviation on first use

Line 11- same as previous point, but for NHS

Materials and Methods

Worth including why focus groups was chosen as the qualitative method.

Elaborating further on what each aspect of EAST means (i.e what the 'easy' aspect means) would be advantageous. It may be helpful to include this as Table 1 instead of the current Table.

Findings

Please include an anonymized participant number for each quote. This allows the reader to appreciate it is not just the voice of a few participants being quoted to illustrate themes.

Reviewer **2**

Name **Gidlow, Christopher**

Affiliation **Staffordshire University, Centre for Sport, Health and Exercise Research**

Date **28-May-2025**

COI **None**

The authors have collected data through focus groups with 45 individuals from Middlesborough who are from 'underrepresented groups'. There is commendable effort to collect qualitative data from a diverse cohort with vary needs (e.g., translation, venue preferences, etc.). The paper is presented in the context of NHS Health Check / and CVD checks for those on a disease register, and reasons why those from these groups tend not to engage.

General points

I have some queries concerns about the framing and undertaking of the study that would need to be addressed to be publishable.

1. Focus. It is unclear to what extent this relates to NHS Health Check, more general health checks or CVD risk assessment. The authors use different terms (NHS CVD Health Check, Annual Health Check). The description in the introduction seems to reflect NHSHC (every 5 years, 40-74 year olds), but various terms are used (and Annual Health Check is used in the title, despite them being every 5 years). This needs to be precise and consistent.

It would also help if the focus group topic guide were appended to see the specificity of questions to NHSHC.

2. Participants. The participants don't appear to have been recruited on the basis of any experience of the NHSHC or CVD risk assessment – why? Migrant doctors no doubt have relevant insights, but there is no justification, and we are unclear as to the extent to which they were briefed on the NHSHC processes etc. in advance of the focus groups. If the authors set out with the aim of exploring NHSHC uptake, why not recruit on that basis – target those invited but who did not attend, or at least on the basis of eligibility. As presented the link between research question/aim and study design and execution feels a little weak.

3. Analysis. I found the description of analysis somewhat confusing. Framework analysis is said to have identified key themes, but then another framework (EAST) was applied. It is

unclear how. What were the themes at the end of the Framework analysis, and were these changed to reach the 4 themes presented in the results? I appreciate that word limits prevent great deal but there is not enough to understand how this was undertaken.

4. Literature. The introduction does not really engage with any of the literature around uptake of NHSHC or similar programmes. This adds to above issue around framing of the study but also fails to make clear what this study will add. There is a little of this in the Discussion but not enough. Of the 19 references, only 11-12 are academic articles, and very few are papers around uptake of NHSHC or similar. Given the abundance of literature, the authors need to do far more to demonstrate engagement with it and to fully contextualise their study and findings. Just a few that are relevant but absent (and there will be many more):

https://bmjopen.bmj.com/content/12/2/e052832.abstract

https://link.springer.com/article/10.1186/s12889-019-7889-4

https://www.rand.org/content/dam/rand/pubs/external_publications/EP60000/EP67129/RAND_EP67129.pdf

https://archpublichealth.biomedcentral.com/articles/10.1186/s13690-015-0064-1

5. Claims. The authors need to check that claims that are either justified on the basis of this study and do not reach beyond (e.g., a need for mass media campaigns) or infer generalisability, despite the case made for this being a geographical and participant sample being very specific to the context.

6. Co-production of interventions. This is not well-defined nor described. It if was part of the study, describe in the methods and results. At present it is mentioned in the discussion, but there needs to be greater clarity in how this sits in relation to the study.

Specific points:

I have not provided a line-by-line list given the more substantive changes above. But some examples:

- Abstract: the first sentence of the Conclusion is generic and does not come from the findings

- Intro Line 10: delete 'significant'

- Line 94 – Focus groups (not focus group interviews)

- There are several uses of 'attrition' where the authors are referring to lack of uptake or non-engagement

- Final para of the Results section belongs in the discussion

- Inclusion criteria should be bullet-pointed and check to improve clarity on exactly how you identified and screened your sample

- Theme 3 – Social aspect – seems to mostly relate to community and/or outreach. Consider renaming.

- Need a cogent definition of ‘under-served’ and how this was operationalised in your study, inclusion criteria etc.

- Qualitative cited as a limitation. It is not. If you set an aim or RQ that demands a qualitative approach, then it is entirely appropriate.

VERSION 1 - AUTHOR RESPONSE

Reviewer: 1
Dr. Katherine Marino, Keele University School of Medicine
Comments to the Author:
Thank you for your work on this project. It is a great topic and well-written paper.

We would like to thank Reviewer 1 for these kind comments on our manuscript. We hope our minor changes improve the manuscript further and they will be in a position to recommend the manuscript for publication.

General Comments
Percentages are best kept to whole numbers, without decimal places, to help the piece read better- personal opinion and will leave this to authors discretion.

Please see changes in percentages reflecting these comments (line 30-33).

Introduction
Good introduction, sets the topic up well.
Line 3- UK shouldn’t be an abbreviation on first use

Please see amendment to United Kingdom (line 3).

Line 11- same as previous point, but for NHS

Please see amendment (line 11).

Materials and Methods
Worth including why focus groups was chosen as the qualitative method.

Please see justification and accompanying reference for the focus group approach (line 66-69).

Elaborating further on what each aspect of EAST means (i.e what the ‘easy’ aspect means) would be advantageous. It may be helpful to include this as Table 1 instead of the current Table.

Please see addition of Table 2 (line 133) to explain the 4 pillars of the EAST framework in relation to our research question/area and topic guide. We hope this is useful to increase reader understanding.

Findings

Please include an anonymized participant number for each quote. This allows the reader to appreciate it is not just the voice of a few participants being quoted to illustrate themes.

Unfortunately, due to poor audio quality and the use of translators and interpreters in some focus groups, we were unable to confidently attribute quotes within transcripts to different participants. However, at the end of each focus group, participants agreed on a summary of what was discussed. We ensured there was no dominant voice, and all participants were able to share and contribute. We ensured a wide range of voices were included in the transcripts. This has been added as a limitation of the study (line 381-386).

Furthermore, we identified in the manuscript which quotes were from which focus groups and ensured to use quotes weighted across all focus groups. I hope this gives the reviewer confidence that we used perspectives and quotations from the full range of participants.

Reviewer: 2
Dr. Christopher Gidlow, Staffordshire University
Comments to the Author:
The authors have collected data through focus groups with 45 individuals from Middlesborough who are from 'underrepresented groups'. There is commendable effort to collect qualitative data from a diverse cohort with vary needs (e.g., translation, venue preferences, etc.). The paper is presented in the context of NHS Health Check / and CVD checks for those on a disease register, and reasons why those from these groups tend not to engage.

We would like to thank Reviewer 2 for these kind comments on our manuscript. We hope our changes improve the manuscript further and they will be in a position to recommend the manuscript for publication.

General points
I have some queries concerns about the framing and undertaking of the study that would need to be addressed to be publishable.

1. Focus. It is unclear to what extent this relates to NHS Health Check, more general health checks or CVD risk assessment. The authors use different terms (NHS CVD Health Check, Annual Health Check). The description in the introduction seems to reflect NHSHC (every 5 years, 40-74 year olds), but various terms are used (and Annual Health Check is used in the title, despite them being every 5 years). This needs to be precise and consistent.
It would also help if the focus group topic guide were appended to see the specificity of questions to NHSHC.

Apologies for the error in our wording in some instances. We have updated the title, abstract and text throughout to ensure it is obvious that the focus of the study was to explore the NHS CVD Health Checks (to be had every 5 years). The topic guide, now attached as supplementary material, asked about health checks in general (not CVD health check specific) initially to understand if participants had experience of any health checks then focused more specifically on the NHS CVD Health checks. This approach is common within qualitative studies, to ask more general questions initially and allow the discussion to flow more organically. We chose to follow this approach for a variety of reasons, including allowing for unprompted data, building rapport, supporting natural conversation flow,

moreover, this approach also allows participants to provide context which supports the validity of the findings (**see: Kvale, S., & Brinkmann, S. 2009, Patton, M. Q. 2015, Rubin, H. J., & Rubin, I. S. 2012,)**

2. Participants. The participants don't appear to have been recruited on the basis of any experience of the NHSHC or CVD risk assessment – why? Migrant doctors no doubt have relevant insights, but there is no justification, and we are unclear as to the extent to which they were briefed on the NHSHC processes etc. in advance of the focus groups. If the authors set out with the aim of exploring NHSHC uptake, why not recruit on that basis – target those invited but who did not attend, or at least on the basis of eligibility. As presented the link between research question/aim and study design and execution feels a little weak.

The aim of the research was to explore the perspectives of disadvantaged groups toward NHS CVD health checks, and not to limit participants to a pool of people who had not attended NHS health checks. To obtain a wider range of perspectives from these groups, we chose a broader recruitment strategy as recruitment of disadvantaged and ethnic minority groups for research is often challenging. Therefore, we decided to use this approach rather than targeting specific sub-groups such as those who have/have not attended health checks. We ensured our recruitment strategy was inclusive and our broad inclusion criteria ensured we captured a greater range of perspectives to be more reflective of the disadvantaged and ethnic minority populations in Middlesborough.

By employing non-specific inclusion and exclusion criteria, we hoped to increase uptake, and capture a wider range of perspectives. Please see addition text added to the manuscript to clarify this on line 89-91.

3. Analysis. I found the description of analysis somewhat confusing. Framework analysis is said to have identified key themes, but then another framework (EAST) was applied. It is unclear how. What were the themes at the end of the Framework analysis, and were these changed to reach the 4 themes presented in the results? I appreciate that word limits prevent great deal but there is not enough to understand how this was undertaken.

We thank the reviewer for pointing this out and we apologise that the analysis section was unclear. We have condensed and reworded this section to help with clarity (line 111-131). We employed a qualitative framework analysis technique, structured according to the EAST framework. In keeping with accepted conventions of qualitative framework analysis, we remained cognisant of any other thematic patterns or trends in the data that could have been of importance to participant experiences and to our overall interpretation of the data (see: Gale, N.K. 2013).

4. Literature. The introduction does not really engage with any of the literature around uptake of NHSHC or similar programmes. This adds to above issue around framing of the study but also fails to make clear what this study will add. There is a little of this in the Discussion but not enough. Of the 19 references, only 11-12 are academic articles, and very few are papers around uptake of NHSHC or similar. Given the abundance of literature, the authors need to do far more to demonstrate engagement with it and to fully contextualise their study and findings. Just a few that are relevant but absent (and there will be many more):
https://bmjopen.bmj.com/content/12/2/e052832.abstract
https://link.springer.com/article/10.1186/s12889-019-7889-4
https://www.rand.org/content/dam/rand/pubs/external_publications/EP60000/EP67129/RAND_EP6

7129.pdf
https://archpublichealth.biomedcentral.com/articles/10.1186/s13690-015-0064-1

Many thanks for suggesting these papers. We have added in more academic literature as references and comparison throughout the introduction (e.g., line 16-20, 34-40) and discussion (e.g., line 306-310, 332-333, 354-356). We hope these additions strengthen the paper, add some contextual information and demonstrate what the study adds to this field of research.

5. Claims. The authors need to check that claims that are either justified on the basis of this study and do not reach beyond (e.g., a need for mass media campaigns) or infer generalisability, despite the case made for this being a geographical and participant sample being very specific to the context.

We have scaled back relevant claims to make them more local-specific, rather than making national recommendations. We agree that the findings are more specific to Middlesborough and may be of more regional relevance and have made recommendations on this scale- see additions through discussion (e.g., line 327-335, 337, 345-346).

6. Co-production of interventions. This is not well-defined nor described. It if was part of the study, describe in the methods and results. At present it is mentioned in the discussion, but there needs to be greater clarity in how this sits in relation to the study.

This study was conducted in collaboration with the Health Innovation Network. As part of the funding requirements of this project, an aim was to co-design policy and practice solutions with communities based on the qualitative findings. We have now added this information to the aims section (line 53-55) and highlighted that as this is an implication of the actual qualitative research study described within the manuscript, it will be referred to within the discussion only (implications for policy and practice section of discussion- line 58-59 and 340-356). We believe that as the co-design is a consequence of the study described in the manuscript, it is not necessary to add information about the co-design elements into the methods and results sections. We hope the reviewer agrees with this reasoning.

Specific points:

I have not provided a line-by-line list given the more substantive changes above. But some examples:
- Abstract: the first sentence of the Conclusion is generic and does not come from the findings

We have amended this sentence to reflect the study conclusions (line 27-28).

- Intro Line 10: delete 'significant'

Please see deletion (line 10).

- Line 94 – Focus groups (not focus group interviews)

Please see deletions throughout.

- There are several uses of 'attrition' where the authors are referring to lack of uptake or non-engagement

Please see amendments throughout (e.g., line 6 and 19).

- Final para of the Results section belongs in the discussion

We have removed the final paragraph of the results and condensed it (to avoid repetition) and incorporated it into paragraph 1 of the discussion (line 272-296).

- Inclusion criteria should be bullet-pointed and check to improve clarity on exactly how you identified and screened your sample

Please see bullet points for inclusion criteria (line 71-88). We have added a sentence regarding screening, to make it clear that there was no need to screen participants due to the community leaders doing the recruitment, which meant they were able to screen potential participants on our behalf (line 90-91).

We have also identified that it is a potential limitation that community leaders may have known participants, adding the potential for bias in the recruitment process – this has been added to the limitations section (line 379-381).

- Theme 3 – Social aspect – seems to mostly relate to community and/or outreach. Consider renaming.

We have kept the 4 pillars of the EAST framework (Easy, Acceptability, Social and Timely) as subheadings for the results, as we conducted framework analysis to group qualitative findings within the 4 pillars of this framework. The authors agree that many of the sub-themes within the "Social aspect" pillar are relevant to community and/or outreach and have added this as a subheading to aid clarification (line 215). We hope this addition is sufficient.

- Need a cogent definition of 'under-served' and how this was operationalised in your study, inclusion criteria etc.

We have added a definition that "underserved groups" will be used as a synonym of people living within Middlesbrough falling within Core20PLUS5 communities (e.g., most deprived 20% of the population, ethnic minority communities, immigrants, refugees and those at risk of certain clinical conditions). We have added this definition in the introduction (line 43-46) and methods inclusion criteria (line 74). We hope this helps with clarity of what is defined as "underserved" within the Middlesbrough population- it is geographically specific based on the Core20PLUS5 protected characteristics of the region. We have changed terminology to refer to the study population as "underserved groups" throughout the manuscript to aid clarity.

- Qualitative cited as a limitation. It is not. If you set an aim or RQ that demands a qualitative approach, then it is entirely appropriate.

Please see removal of the qualitative study design as a limitation (line 386-390). We apologise for overlooking this previously - this limitation was meant to suggest that the addition of a quantitative element may allow researchers to "study if barriers and facilitators have differing importance amongst different groups".
